# Dynamic automated assessment of functionally limited temporomandibular disorders using motion analysis

**DOI:** 10.1177/03000605241303190

**Published:** 2024-12-14

**Authors:** Ruibin Wu, Yuzhen Jiang, Yinghui Zhu, Wei Wang, Peizhu Huang, Xiaoqing Dong, Muhammad Suhail Shaikh

**Affiliations:** 1 Chaozhou People’s Hospital, Chaozhou, Guangdong Province, China; 2 Hanshan Normal University, Chaozhou, Guangdong Province, China

**Keywords:** Functionally limited, temporomandibular disorder, temporomandibular joint, assessment, automatic detection, motor function analysis

## Abstract

**Objective:**

This study investigated a method for assessing movement patterns in patients with functionally limited temporomandibular disorders using minimal equipment. The aim was to enable clinicians to better understand temporomandibular joint function, support treatment decision-making, and provide data for documenting disease progression.

**Methods:**

This retrospective, observational study included 45 patients with functionally limited temporomandibular disorders (Group A) and 40 healthy volunteers (Group B). Video data of temporomandibular joint movements were recorded. Measurements, including maximum mouth opening distance, maximum offset distance, maximum offset angle, direction of offset, and fluctuation graphs of offset angle and distance, were obtained using manual methods and an automatic measurement program. The results were statistically analyzed.

**Results:**

Statistically significant differences were observed between patients and the control group in the manually measured mouth opening distance and offset distance, as well as in the automatically measured offset distance and offset angle.

**Conclusion:**

Automated testing methods can effectively assess the motor function of functionally limited temporomandibular joints, providing more objective, data-driven, and graphical testing indexes. Further research is warranted to explore their practical significance.

## Background

Temporomandibular disorders (TMDs) are clinically characterized by joint rattling, joint pain, jaw dysmobility, earache, toothache, and headache. They affect up to 15% of the adult population,^1^ with a peak incidence in patients’ 20s and 40s. Prolonged exposure to this condition can lead to psychological anxiety and depression. Among TMDs, functional limitations play a significant role, and accurate assessment of mouth limitations and deviations is critical in determining the need for joint loosening treatment and identifying the tissue structure, direction, and strength required for manual therapy.

Computed tomography (CT) scans^2,3^ and three-dimensional (3D) CT scans^4^ are available to analyze the relationship between the condylar fossa and the condyle in patients with TMDs, while magnetic resonance imaging (MRI) can produce 3D models.^5^ Klatkiewicz et al.^6^ advocate ultrasonography as a standard diagnostic procedure for TMDs because of its rapid examination time, low cost, and noninvasive nature. Additionally, 3D fluoroscopy allows for quantitative evaluation of the mandible’s *in vivo* 3D mobility and associated endpoint trajectories.^7^ Other studies have examined the complex biomechanics of jaw movement muscles.^8^ Many devices have been used in the study of TMDs, including infrared cameras,^9^ surface electromyography,^10^ mandibular kinematics,^11^ motion decoders,^12^ opening force measurement devices,^13^ 3D electromagnetic arthrograms,^14^ motion analyzers,^15^ 3D ultrasonic jaw motion analyzers,^16^ and optoelectronic devices and photo capture systems.^9,17–19^

Assuming that we will perform joint release manipulation on a patient with functionally limited temporomandibular joint (TMJ) dysfunction, how can we evaluate the functional limitation and document the progression of the disease? In clinical practice, we identified certain limitations in commonly used diagnostic methods. The manual detection method, which is widely used, is subject to examiner bias in the evaluation of TMJ dysfunction. Similarly, CT bone 3D reconstruction and MRI examination methods have static limitations because they cannot display motor pattern disorders. Existing research protocols also have some shortcomings and often involve unnecessary complexity. For instance, one research group proposed a tool using low-cost, simple, noninvasive, and flexible methods.^20^ However, this method still requires securing a marker intraorally through a connecting rod, which interferes with physiological activities and makes the procedure somewhat cumbersome.

This study aims to provide a simpler method for observing the structural and functional aspects of the TMJ to detect and evaluate its motor-functional condition without requiring complicated equipment. This approach allows clinicians to better understand changes in joint movement, the degree of offset, and other observational indicators. These insights can aid in decisions about adding joint mobilization and adjusting the direction and range of motion during manipulation, as well as in recording disease progression in the form of data.

The assay proposed in this study evaluates TMJ function by examining mandibular movement. The TMJ consists of the condyle, articular fossa, articular disc, ligaments, and muscles, with the left and right sets of structures forming a kinematic joint system that drives mandibular movement. Disorders in the mechanical and dynamic structures of the TMJ result in changes to mandibular movement, which in turn reflect the function of the TMJ. This principle forms the basis for studying the function of the TMJ.

## Materials and methods

### Patients

This retrospective observational study involved patients who visited the Department of Orthopedics and Traumatology at Chaozhou People’s Hospital between January 2020 and July 2022. The three inclusion criteria were as follows: all patients were diagnosed using the diagnostic criteria for TMDs^21^ and presented with limited mouth opening or distorted open faces, were able to cooperate with the recording of the entire TMJ mouth opening and closing process, and had clear recorded video material. The exclusion criteria included rheumatism, rheumatoid disease, dentofacial problems, orthodontic treatment, dental problems, and a history of surgery; a history of psychiatric disorders; and complex coloration of the video material's background pattern, which interfered with program recognition.

Forty-five patients with functionally limited TMD were enrolled, and 37 healthy volunteers were recruited as a control group during the same time period. The Ethics Committee of Chaozhou Municipal People’s Hospital, Guangdong Province, China approved the study protocol (Approval No. CZSRMYY-20200113001; January 13, 2020). This study adhered to the 1975 Declaration of Helsinki (updated in 2013). All patients and volunteers provided written informed consent, and their information was de-identified and kept confidential.

## Study protocol

The reporting of this study conforms to the STROBE guidelines.^22^

### Manual measurement method

The maximum anterior incisal midline opening distance and the maximum incisal midline excursion distance were manually measured with a ruler when the natural mouth opening was at its maximum.

### Automatic measurement equipment and methods

**Figure 1.**
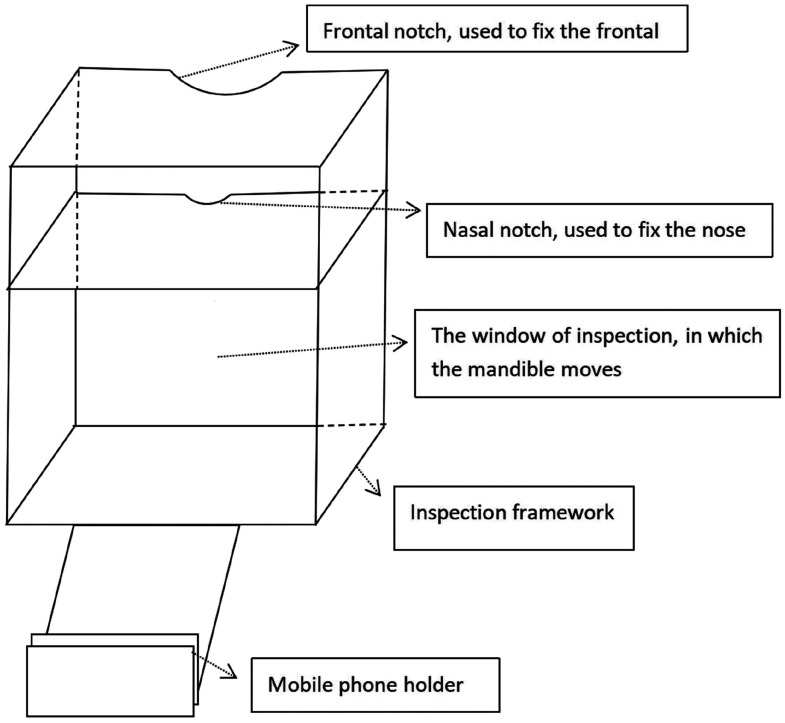
Dynamic image acquisition device for the TMJ.

Our team designed an image acquisition device (Figure 1) to record video data on the dynamics of the patients’ TMJs, along with supporting application software to automatically identify pre-marked marker points and extract data indicators such as maximum mouth opening distance and maximum offset distance. The specific operation process was as follows. White marking points (5-mm white stickers) were applied at the patient’s nasolabial groove midpoint and the depression of the chin–labial groove. The bridge of the nose and the forehead were tightly pressed into the traces of the checking device. The head was fixed to maintain stability, avoiding movement, and the camera device was focused and locked. Dynamic image data of the patient’s TMJ movement were then recorded. The recorded data were analyzed using software that automatically measured the maximum movement distance, maximum deviation distance, maximum deviation angle, and direction of deviation before and after the mouth was opened with the marking points.

**Figure 2.**
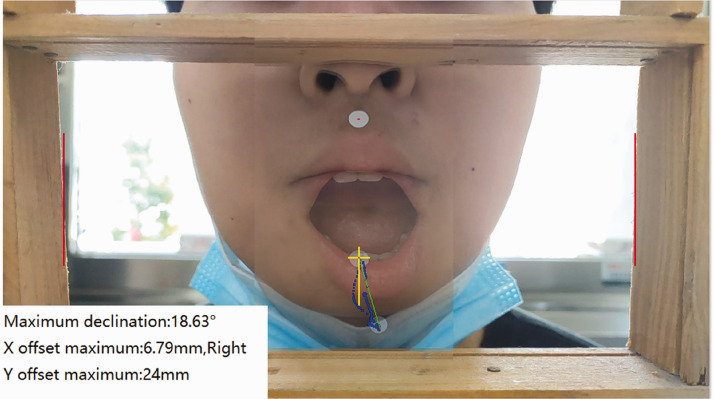
The graphic depicts the patient’s video frame overlap before and after the mouth reaches its maximum size, as well as the measurement data in the lower left corner.

The automatic detection measurement method was performed as follows. The video frame diagram was obtained before mouth opening and at its maximum. The overlap of the two drawings was used to determine the characteristic values of the white points before and after the opening. The white point on the upper lip was designated point A, and its position at maximum opening was designated point A′. Similarly, the white point on the lower lip was designated point B, and its position at maximum opening was designated point B′. The vertical distance between points B and B′ was recorded as the maximum mouth opening distance, the horizontal distance was recorded as the offset distance, and the angle between line B and A′B′ was recorded as the offset angle (Figure 2). The software algorithm determined the positions of the left and right inner edges of the border, marked these with a red vertical line, and identified the center positions of the two white marking points on the upper and lower lips in the initial video frame. It then tracked the center positions of these marking points across each frame of the video and identified their positions in the closed state. The white point data were analyzed to extract feature values, which were displayed as image data.

## Statistical methods

The measurement results were organized and summarized, and data such as the opening distance, offset distance, and offset angle were recorded to establish a database. Statistical analyses and chart generation for the screening data were performed using IBM SPSS Statistics for Windows, Version 26.0 (IBM Corp., Armonk, NY, USA) and GraphPad Prism 9.0 software. The measurement data were tested for normality, and those conforming to a normal distribution were expressed as mean ± standard deviation. An independent sample t-test was used for data analysis. Data not conforming to a normal distribution were described using medians and quartiles and were analyzed using nonparametric tests. A *P*-value of <0.05 was considered statistically significant.

## Results

1. The study included 45 patients with functionally limited TMDs (Group A) and 40 healthy volunteers (Group B). One of the 45 patients with functionally limited TMD did not meet the inclusion criteria and was excluded from the study. The remaining 44 patients were registered, consisting of 24 male and 20 female patients aged 12 to 68 years (mean age, 29.6 ± 14.8 years). Similarly, 3 of the 40 healthy volunteers did not meet the inclusion criteria and were excluded. The remaining 37 healthy volunteers were enrolled, consisting of 29 male and 8 female patients aged 13 to 70 years (mean age, 34.1 ± 16.3 years).
2. The maximum mouth opening distance was measured manually. In Group A, the mean maximum opening distance was 25.64 ± 1.09 mm, whereas that in Group B was 29.84 ± 0.80 mm. The mean difference was 4.20 ± 1.40 mm, with a 95% confidence interval (CI) of 1.41 to 6.99 mm (*P* = 0.0036). This difference was statistically significant, as shown in Figure 3(a). The largest offset distance was also measured manually. In Group A, the mean offset distance was 4.39 ± 0.26 mm, whereas that in Group B was 1.38 ± 0.12 mm. The mean difference was −3.0 ± 0.31 mm, with a 95% CI of −3.63 to −2.39 mm (*P* < 0.0001). This difference was also statistically significant, as shown in Figure 3(b).

**Figure 3.**
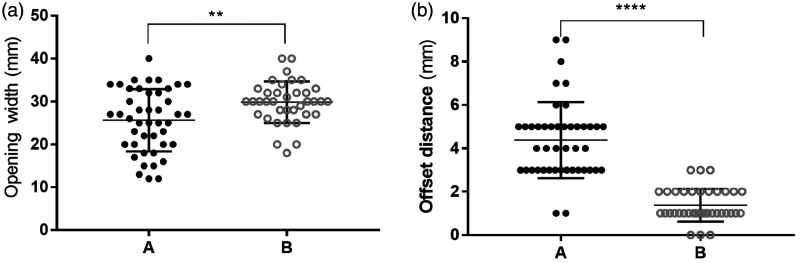
The maximum mouth opening distance and the maximum offset distance were measured by manual measurement methods in patients with functionally limited TMD and normal controls. (a) Maximum opening distance and (b) maximum offset distance. **P < 0.01, ****P < 0.0001.
3. The maximum mouth opening distance was determined automatically. In Group A, the mean maximum opening distance was 23.33 ± 0.99 mm, whereas that in Group B was 24.70 ± 0.94 mm. The mean difference was 1.36 ± 1.38 mm with a 95% CI of −1.38 to 4.11 mm. The difference was not statistically significant, as shown in Figure 4(a). The maximum offset distance was determined automatically. In Group A, the mean offset distance was 3.81 ± 0.25 mm, whereas that in Group B was 2.48 ± 0.18 mm. The mean difference was −1.33 ± 0.32 mm with a 95% CI of −1.96 to −0.70 mm (*P* < 0.0001). The difference was statistically significant, as shown in Figure 4(b). The largest offset angle was determined automatically. In Group A, the mean offset angle was 15.09° ± 1.13°, whereas that in Group B was 8.85° ± 0.78°. The mean difference was −6.24° ± 1.43° with a 95% CI of −9.08° to −3.40° (*P* < 0.0001). The difference was statistically significant, as shown in Figure 4(c).

**Figure 4.**
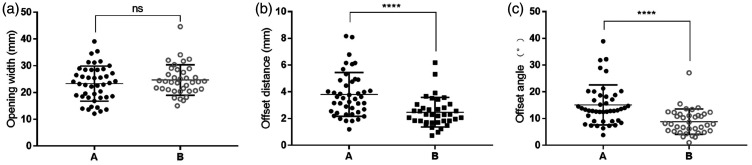
The parameters were measured in patients with TMD and normal controls using an automated assay. (a) Automatic detection of maximum opening distance. (b) Automatic detection of maximum offset distance and (c) automatic detection of maximum offset angle. ns: not significant. ****P < 0.0001.
4. The automatic detection method was used to determine the offset direction, and there was no significant difference between left and right deviation when comparing Groups A and B, as shown in Figure 5.

**Figure 5.**
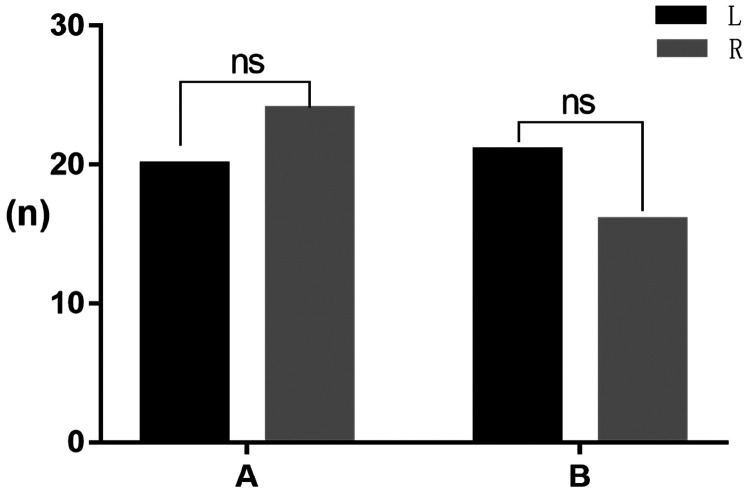
Automatic detection of offset direction. ns: not significant.
5. Fluctuating curves of the TMJ mouth opening distance and offset angle were plotted. The offset angle fluctuated during mouth opening and intensified late in the closing process. The offset distance also fluctuated during mouth opening, swung back later in the closure process, and then closed completely, as shown in Figure 6(a). Larger fluctuations in the angle and distance of fluctuation in the figures indicated poorer stability of the joint movement, while smoother fluctuations indicated better stability of the joint movement, as shown in Figure 6(b).

**Figure 6.**
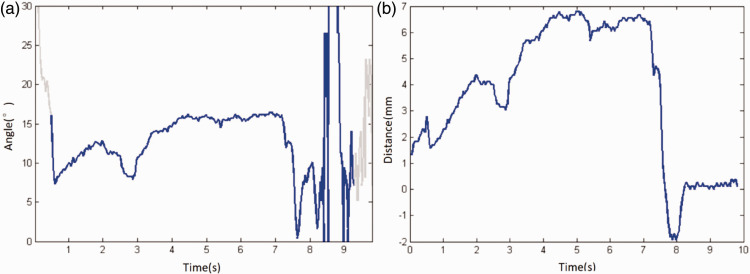
Fluctuation diagrams of TMJ movement stability. (a) Offset angle fluctuation diagram and (b) offset distance fluctuation diagram.

## Discussion

### Characteristics of image recording tools

Several tools are available for recording mandibular movements. Shu et al.^23^ recorded mandibular movements using an optical tracking device. Another study used a motion capture system to record mouth-opening movements based on a CT-constructed model of the mandible and maxilla and an MRI-based model of the TMJ discs.^24^ Furtado et al.^9^ conducted research using three infrared cameras and nine reflecting markers. Piehslinger et al.^25^ captured TMJ jaw-opening movements using a computer and electronic signals monitored by a mandibular position indicator, which they concluded was a useful and practical orthopedic diagnostic tool for assessing the mandible’s ability to rotate and translate. Mapelli et al.^17^ recorded the 3D coordinates of mandibular intertrochanteric and condylar reference points using an optoelectronic motion analyzer. Mazzetto et al.^16^ used digital calipers and a 3D ultrasonic jaw motion analyzer to measure jaw motion. To record TMJ jaw-opening activity, Lezcano et al.^14^ used a 3D electromagnetic arthrocentrometer with a transducer. da Cunha et al.^26^ captured 3D pictures of jaw motions using an infrared camera. Shichita et al.^27^ used six cameras to capture their data. By contrast, the current study utilized more practical and simpler equipment, relying on a cell phone for camera recording. This approach is designed to make the study’s findings more accessible and applicable in clinical practice compared with complex technologies.

### Design and placement of markers for mandibular movement analysis

In this investigation, white markers were placed on the skin at the midpoints of the nasolabial sulcus and the depression of the chin–labial sulcus. The oral vestibule inside these two locations is maintained by the frenum of the upper and lower lips, and the relative displacement of the skin and bone during mandibular movement is minimal. Therefore, marker activity can provide a relatively accurate representation of mandibular activity. Lezcano et al.^14^ placed sensors within the lower lip on the interincisional median line of the mandible to serve as markers for localization and to record mandibular movement. da Cunha et al.^26^ designed markers made of stainless steel wires and spheres covered with reflective material, and Wang et al.^28^ used three fluorescent markers on the upper dentition and four fluorescent markers on the lower dentition to study mandibular opening and closing movements. All of these strategies aimed to derive mandibular movement features from body surface markers to infer and understand TMJ motor activity.

### Standardizing natural mouth opening measurements in detection methods

This study evaluated natural mouth opening indicators rather than the deliberate extreme mouth opening index. The natural mouth opening method was chosen because the joint movement amplitude used in daily life is not typically at its extreme range. According to Türp et al.,^29^ activities such as chewing, talking, oral hygiene, and even yawning do not require very large openings or significant lateral or prominent shifts. They proposed thresholds for impaired jaw function to protect patients and non-patients from medicalization, overdiagnosis, and overtreatment. Khare et al.^30^ asked volunteers to open their mouths as wide as possible. They found that the mean maximal mouth opening was 51.3 ± 8.3 mm in men and 44.3 ± 6.7 mm in women. By contrast, our study did not require patients to achieve their maximum mouth opening. Instead, natural mouth opening was measured, with results showing a width of 25.64 ± 1.094 mm (n = 44) in the patient group and 29.84 ± 0.7984 mm (n = 37) in the healthy group. Türp et al.^29^ analyzed data from 500 patients with TMD at the University of Basel Dental Center and reported mean maximum values for unassisted mandibular openness, protrusion, and left-right movement as 49.3 ± 9.1 mm, 8.8 ± 2.3 mm, 9.4 ± 2.5 mm, and 11.8 ± 3.1 mm, respectively.

### Detection indicators

The automated measurements in this study determined parameters such as mouth opening distance, deflection distance, deflection angle, and deflection direction. Shu et al.^24^ used an optical tracking system to collect and analyze the trajectory of mouth opening movements and to quantify stress changes at various stages (particular trajectories). da Cunha et al.^26^ found that any type of temporomandibular dysfunction exhibited higher lateral deviation or obliquity in opening or closing movements. Andrade et al.^31^ proposed a quantitative assessment of asymmetry in mandibular opening and closing movements by evaluating the degree of irregular movements. Türp et al.^29^ measured the maximum values of mandibular openness, protrusion, and side-to-side movements to quantify mandibular mobility. Although the description and analysis of graphic recordings of jaw and TMJ movements provide an indirect understanding of TMJ function, they remain a valid means of assessing whether TMJ function is affected. Marpaung et al.^18^ investigated the validity of functional examinations following TMJ disc displacement, concluding that disc displacement is clinically relevant only when it interferes with TMJ function and recommending that functional diagnostic methods be prioritized. In clinical practice, this study team pre-tested and determined each patient’s mouth opening, offset distance, and offset direction before performing joint release exercises. These parameters formed the foundation for therapeutic decisions, guiding which side of the myofascial joint capsule and other tissues required relaxation through manipulative therapy, as well as determining the force and direction of manipulation. This highlights the importance of investigating these indicators in assessment and treatment.

### Automatic detection and manual detection

The traditional motor function assessment of patients with TMD using manual measurement methods has limitations. Manual inspection makes it difficult to capture detailed and accurate joint movements because of the subtle changes in joint movement and the inherent subjectivity and variability of manual measurements. By contrast, intelligent recognition technology digitizes and preserves the process of joint movement, transforming subjective and non-archival evaluations into digital archives that enable traceability and the analysis of data changes throughout the treatment process. Automatic testing evaluates the patient’s TMJ function, providing objective and quantitative indicators that clinicians can use as a basis for diagnosis and treatment. These indicators also allow clinicians to reference original data when evaluating treatment efficacy and reviewing data changes during rehabilitation. Compared with manual testing, automatic testing is more objective, credible, and reliable.

### Application and research stage of dynamic intelligent recognition

The rapid development of image recognition, face recognition, and feature graphic recognition technologies across various fields provides a strong foundation for advancing this method of examination. There is a growing demand for more reasonable and objective approaches to evaluating diseases and generating records. The current interdisciplinary collaboration among team members aims to address the dynamic examination of TMJ movement function, enabling clinicians to better understand changes in joint motion and the degree of deviation, thereby facilitating the creation of more personalized treatment plans.

The application of dynamic intelligent identification allows the recognition of mouth opening deviation distances and angles, quantifying these indices to help physicians adjust treatment plans based on the results. This approach holds significant therapeutic value. Additionally, it was observed that poor TMJ movement stability results in greater oscillations during movement. Future research will focus on developing methods to assess the stability of joint movement.

Notably, this study is limited by the use of two-dimensional video images, which present depth-of-field issues that affect the accuracy of automatic open-mouth distance measurements. The availability of 3D dynamic video specimens would address this limitation. Another challenge involves the need for a standardized screening system, including appropriate hardware, standardized screening postures, access to standardized patient samples, and the development of robust identification software.

## Conclusions

Intelligent recognition technology can identify the offset distance, offset angle, and offset direction in patients with TMD. It can also generate fluctuation curve diagrams for offset angle and direction during joint movement, providing clinicians with a more comprehensive understanding of the disorder’s functional state and more reliable data to guide treatment decisions and monitor disease progression. However, the depth-of-field issue remains unresolved, which limits the accuracy of the automatic identification of mouth opening distance. To overcome this limitation, future studies will need to incorporate 3D dynamic video specimens.

## Data Availability

The datasets used and/or analyzed during the current study are available from the corresponding author on reasonable request.
